# Clinical spectrum and prognostic factors of possible UIP pattern on high-resolution CT in patients who underwent surgical lung biopsy

**DOI:** 10.1371/journal.pone.0193608

**Published:** 2018-03-28

**Authors:** Yasuhiro Kondoh, Hiroyuki Taniguchi, Kensuke Kataoka, Taiki Furukawa, Ayumi Shintani, Tomoyuki Fujisawa, Takafumi Suda, Machiko Arita, Tomohisa Baba, Kazuya Ichikado, Yoshikazu Inoue, Kazuma Kishi, Tomoo Kishaba, Osamu Nishiyama, Takashi Ogura, Keisuke Tomii, Sakae Homma

**Affiliations:** 1 Department of Respiratory Medicine and Allergy, Tosei General Hospital, Seto, Japan; 2 Department of Respiratory Medicine, Nagoya University Graduate School of Medicine, Showa-ku, Nagoya, Aichi, Japan; 3 Department of Medical Statistics, Osaka City University Graduate School of Medicine, Osaka, Japan; 4 Second Division, Department of Internal Medicine, Hamamatsu University School of Medicine, Hamamatsu, Japan; 5 Department of Respiratory Medicine, Kurashiki Central Hospital, Okayama, Japan; 6 Department of Respiratory Medicine, Kanagawa Cardiovascular and Respiratory Center, Yokohama, Japan; 7 Division of Respiratory Medicine, Saiseikai Kumamoto Hospital, Kumamoto, Japan; 8 Clinical Research Center, National Hospital Organization Kinki-Chuo Chest Medical Center, Osaka, Japan; 9 Department of Respiratory Medicine, Respiratory Center, Toranomon Hospital, Tokyo, Japan; 10 Department of Respiratory Medicine, Okinawa Chubu Hospital, Uruma, Okinawa, Japan; 11 Department of Respiratory Medicine and Allergology, Faculty of Medicine, Kindai University, Osaka, Japan; 12 Department of Respiratory Medicine, Kobe City Medical Center General Hospital, Kobe, Japan; 13 Department of Respiratory Medicine, Toho University Omori Medical Center, Tokyo, Japan; Keio University, JAPAN

## Abstract

**Background:**

Few studies have reported the diagnostic variability in patients with a possible usual interstitial pneumonia (UIP) pattern on high-resolution CT (HRCT) who underwent surgical lung biopsy (SLB), and the prognostic factors for these patients have not been fully evaluated. We retrospectively investigated the frequency of idiopathic pulmonary fibrosis (IPF) and prognostic factors in patients with possible UIP pattern on HRCT.

**Methods:**

Consecutive patients who had a possible UIP pattern on HRCT, underwent SLB, and had a diagnosis of IIPs before SLB were retrospectively recruited from 10 hospitals. Diagnoses were made based on multidisciplinary discussion using the criteria for current IPF guidelines and multidisciplinary classification for IIPs in each hospital.

**Results:**

179 patients who underwent SLB were enrolled. The diagnoses were IPF in 91 patients (51%), unclassifiable IIPs in 47 (26%), idiopathic NSIP in 18 (10%), and chronic hypersensitivity pneumonia in 17 (9%). One-year FVC changes showed significant differences between IPF and non-IPF (-138.6 mL versus 18.2 mL, p = 0.014). Patients with IPF had a worse mortality than those with non-IPF (Logrank test, p = 0.025). Multivariable Cox regression analysis demonstrated that diagnoses of IPF (HR, 2.961; 95% CI, 1.183–7.410; p = 0.02), high modified MRC score (HR, 1.587; 95% CI, 1.003–2.510; p = 0.049), and low %FVC (HR, 0.972; 95% CI, 0.953–0.992; p = 0.005).

**Conclusions:**

About a half of patients with a possible UIP pattern on HRCT had diagnoses other than IPF, and patients with IPF had a worse mortality than those with an alternative diagnosis. We reaffirmed that multidisciplinary discussion is crucial in patients with possible UIP pattern on HRCT.

## Introduction

Idiopathic pulmonary fibrosis (IPF) is an intractable disease with a poor prognosis. Therefore, the accuracy of a diagnosis of IPF is crucial for making therapeutic decisions and predicting patient’s survival [1,2]. In recent years, diagnosis with a high resolution CT (HRCT) has played a central role in the diagnosis of interstitial lung disease (ILD), and the IPF guidelines of the American Thoracic Society (ATS), the European Respiratory Society (ERS), the Japanese Respiratory Society (JRS) and the Latin-American Thoracic Society (ALAT) in 2011 say that a diagnosis of IPF can be made with a usual interstitial pneumonia (UIP) pattern on HRCT [2]. Meanwhile, patients presenting a possible UIP pattern on HRCT require a surgical lung biopsy (SLB) for a definitive diagnosis of IPF.

Antifibrotic drugs, pirfenidone and nintedanib, are now regarded as “conditional” recommendation drugs for IPF [3–9], so an early detection of IPF in ILD is essential in providing clinical guidance for a treatment of IPF. Fell et al. studied factors associating with a diagnosis of IPF among patients with ILD cohort who underwent SLB [10]. According to their study, a majority of patients with a possible UIP pattern on HRCT can be diagnosed with a high degree of certainty as having IPF with a pathological UIP pattern, especially if the individual is in the older age group [11].

Following Fell’s study, Raghu et al. assessed patients with SLB specimens that were centrally screened for inclusion in the ARTEMIS-IPF trial, and reported that a possible UIP pattern on HRCT had a positive predictive values (PPV) of 94% (79/84) for the presence of a pathological pattern of UIP [12]. Yagihashi et al. studied the patients enrolled in three studies sponsored by the Idiopathic Pulmonary Fibrosis Clinical Research Network (IPFnet) at 26 sites throughout the USA *(Prednisone*, *Azathioprine*, *and N-Acetylcysteine*: *a Study that Evaluates Response in Idiopathic Pulmonary Fibrosis (PANTHER-IPF)*, *Sildenafil Trial of Exercise Performance in Idiopathic Pulmonary Fibrosis (STEP-IPF)* and *Anticoagulant Effectiveness in Idiopathic Pulmonary Fibrosis (ACE-IPF))*, and reported that among 64 patients with possible UIP pattern on HRCT, 60 patients had a pathological UIP pattern and 4 patients had a pathological probable UIP pattern [13]. On the basis of these findings, a possible UIP pattern on HRCT has been considered sufficient to make a diagnosis of IPF in clinically appropriate settings [12]. However, because the patients with possible UIP pattern in the latter analyses were taken from clinical trials and only those with IPF diagnosed by site investigators with SLB interpretation (ie, patients thought to have UIP from SLB) [12,13] were included while those with conditions other than IPF based on site evaluations were inevitably excluded, we suspect that the results of such populations differ from those of the general population in clinical practice, and are highly biased. In addition, the PPV may be higher in a highly selected cohort that contains a high prevalence of IPF, and may be lower in an ILD cohort which may contain several diseases other than IPF.

A recent paper by Brownell et al. reported that only 60.9% (39 in 64) of patients with possible UIP pattern on HRCT had a diagnosis of IPF in the UCSF cohort but in the Mayo cohort the percentage was 97% (69 in 71) [14]. Therefore, at present little is known about the diagnostic variability and the clinical impact on survival of that variability in cohorts of ILD patients who have a possible UIP pattern on HRCT and undergo surgical lung biopsy. Therefore, we aimed to assess the disease frequencies, the PPV for IPF, and prognostic factors in a large cohort of biopsy-proven ILD patients with a possible UIP pattern on HRCT who did not have known causes of ILD, such as connective tissue disease (CTD) or chronic hypersensitivity pneumonitis (CHP), and had a possibility of a diagnosis of IPF before SLB.

## Materials and methods

### Study subject

This study was a retrospective, exploratory, multi-center study. Patients who underwent initial evaluation from March 2006 to March 2015 were recruited consecutively during the enrollment period from June 2015 to October 2015. Patients who had a possible UIP pattern on HRCT, underwent SLB, and had a diagnostic possibility of IPF before SLB were eligible if there were ten or more patients in a fixed period at each institution. Diagnosis of a possible UIP pattern was made by a site investigator according to the diagnostic criteria of the 2011 IPF guidelines [2] in the enrollment period.

We excluded patients with the following: 1) diagnosis of UIP pattern or inconsistent with UIP pattern on HRCT; 2) satisfaction of the diagnostic criteria for concurrent lung cancer, specific ILDs other than idiopathic interstitial pneumonias (IIPs), generalized systemic disease, or CTD before SLB (cases that did not fulfill these diagnostic criteria were not excluded even when there were physical or blood findings indicative of CTD. Cases in which CTD developed during the follow up period were also not excluded); or 3) other reasons for which a physician participating in the study judged a patient to be unsuitable.

### Data collection

Clinical data were obtained retrospectively from patient records (S1 Dataset). The diagnosis at the enrollment period was made through multidisciplinary discussion (MDD) using the criteria for 2011 IPF guidelines and multidisciplinary classification 2013 for IIPs in each hospital. The initial diagnoses before 2013 were reevaluated in the enrollment period. The final diagnoses were used for the diagnostic evaluations. A diagnosis of “unclassifiable idiopathic interstitial pneumonia” was applied using the criteria in the 2013 multidisciplinary classification for IIPs [15]. A broader pathological UIP pattern, which was defined as a UIP pattern and additional pathological patterns suggestive but not definitive for a diagnosis of CTD (e.g., lymphoid aggregates with germinal center and/or prominent plasmacytic infiltration) or CHP (e.g., centrilobular and/or bridging fibrosis), or a pattern in which differentiation between a UIP pattern and a NSIP pattern was difficult, was evaluated. We evaluated patient characteristics and pulmonary function tests, PaO_2_, bronchoalveolar lavage fluid (BALF) and serologic test results conducted within a month before biopsy. The modified Medical Research Council (MRC) scale was used to evaluate dyspnoea in daily living.

One-year (± 3 months) follow-up data on forced vital capacity (FVC) were obtained, and the annual rate of decline in FVC (measured in milliliters per year) was studied. Survival status with mortality was evaluated in June, 2016.

Because of the anonymous nature of the data, the requirement for informed consent was waived. The study was approved by the Tosei General Hospital institutional ethics committee (IRB No. 494–1).

### Statistical analysis

Data are presented as mean ± standard deviation or median (range), as appropriate. We used t-tests and Man-Whitney U test to compare the averages of continuous variables (such as age) and chi-square tests to compare proportions of categorical variables (such as gender) between groups. We examined PPVs, specificities, sensitivities, and negative predictive values (NPVs) when classifying patients with IPF in possible UIP pattern patients on HRCT for each age category.

Univariate and multivariable Cox proportional hazards regression analyses were performed to evaluate the relationship between each variable and the mortality with adjustment for age and gender. The Harrell’s C statistic was used to investigate the capability of each Cox proportional hazards regression model to predict mortality. Cumulative probabilities of survival were plotted with Kaplan-Meier method, and were compared by the log rank test. All data were analyzed using a statistical software package (SPSS, version 23.0; SPSS, Inc.; Chicago).

## Results

### Diagnosis and baseline characteristics of the study subjects

A total of 179 patients from 10 hospitals were enrolled. The initial and final multidisciplinary diagnoses are shown in Table 1. The final diagnoses were IPF in 91 (50.8%) and non-IPF in 88 (49.2%), unclassifiable IIPs in 47 (26.5%), idiopathic non-specific interstitial pneumonia (idiopathic NSIP) in 18 (10.1%), CHP in 17 (9.5%), and CTD-ILD in 6 (3.4) (Table 1). The diagnosis of 19 of the 179 patients (10.6%) changed from the initial diagnosis. The reasons for unclassifiable IIP were inadequate clinical, radiological, or pathological data in 3 cases, and major discordance between clinical, radiological, and pathological findings in 43 cases in the following situations: (a) new entity, or unusual variant of a recognized entity, not adequately characterized by the current criteria for IIPs in 5; and (b) multiple pathological patterns in 38 (S1 Table). Forty in 47 cases fit a broader definition of pathological UIP pattern (S1 Table). The most suspected diagnoses were CHP in 18, IPF in 12, CTD-ILD in 9, smoking related ILD in 2, and undetermined in 1 (Table A in S1 File).

**Table 1:** Multidisciplinary diagnosis of 179 patients in the cohort.

		Final Diagnosis					
		IPF	Unclassifiable IIPs	NSIP	CHP	CTD-ILD	Total
Initial Diagnosis	IPF	91	2	0	6	2	101
	Unclassifiable IIPs	0	42	0	2	0	44
	NSIP	0	3	18	0	4	25
	CHP	0	0	0	9	0	9
	CTD-ILD	0	0	0	0	0	0
	Total	91	47	18	17	6	179

CTD diagnosis after surgical lung biopsy at registry included

Abbreviations: IPF, idiopathic pulmonary fibrosis; IIP, idiopathic interstitial pneumonia; NSIP, non-specific interstitial pneumonia; CHP, chronic hypersensitivity pneumonitis; CTD-ILD, connective tissue disease-interstitial lung disease

Baseline characteristics in IPF and non-IPF showed no significant differences except in BALF % macrophages (87.8% vs 82.0%, p = 0.014) and neutrophils (1.0 vs 2.0, p = 0.025) (Table 2).

**Table 2:** Baseline characteristics of possible UIP pattern on HRCT: Comparison between IPF and non-IPF.

	Total	IPF	Non-IPF	p value	n
Age, y.o.median (IQR)	65 (60–70)	65 (60–70)	65 (59–70)	0.735	179
Gender, male	123	67	56	0.150	179
Smoking, current/ex/never	18/98/63	9/54/28	9/44/35	0.417	179
Modified MRC, 0/1/2/3/4	83/65/20/5/1	45/29/11/4/1	38/36/9/1/0	0.525	174
Cough, yes	124	65	59	0.307	179
FVC, % predicted (Mean±SD)	84.9±20.0	85.2±20.2	84.5±20.0	0.800	179
DLco, % predicted(Mean±SD)	72.5±24.6	73.0±24.1	72.0±25.2	0.787	161
FEV1/FVCX100(Mean±SD)	83.0±7.6	83.0±7.7	83.0±7.7	0.955	179
PaO2, Torr median (IQR)	85.3 (76.5–92.9)	85.6 (76.1–91.5)	84.7 (77.0–94.2)	0.599	174
BAL: Macrophages, % median (IQR)	85 (72.0–94.5)	87.8 (76.0–95.0)	82.0 (65.2–93.0)	0.014	149
Neutrophils, % median (IQR)	1.5 (0.2–4.0)	1.0 (0.0–2.9)	2.0 (0.7–4.3)	0.025	149
Lymphocytes, % median (IQR)	9.2 (2.5–20.1)	8.0 (2.0–16.0)	12.0 (3.9–23.3)	0.054	149
Eosinophils, % median (IQR)	0.6 (0.0–1.7)	0.6 (0.0–1.6)	0.9 (0.0–2.0)	0.342	149

MRC, Medical Research Council scale; FVC, forced vital capacity; DLCO, diffusing capacity for carbon monoxide; FEV1, forced expiratory volume in one second; BAL, bronchoalveolar lavage; SD, Standard Deviation; IQR, Inter-quartile range

### PPVs for a diagnosis of IPF and age

PPVs for a diagnosis of IPF were less than 55 years old (y.o.). in 39.1%, 55 to 64 y.o. in 55.0%, and greater than 64 y.o. in 51.0%, respectively (Table 3, Table B and C in S1 File)(Fig A in S1 File).

**Table 3:** Positive predictive value, negative predictive value, sensitivity, and specificity when classifying patients with IPF based on being at least as old as the age indicated.

Age	IPF	Non-IPF	Total	PPV	NPV	Sensitivity	Specificity	Odds
-54	9	14	23	39.1% (21.1–59.4)	45.4% (37.9–53.1)	9.2% (4.5–15.9)	84.1% (75.5–90.7)	0.535 (0.219–1.305)
55–65	33	27	60	55.0% (42.4–67.2)	51.3% (42.3–60.1)	36.3% (26.9–46.4)	69.3% (59.2–78.3)	1.285 (0.690–2.396)
65-	49	47	96	51.0% (41.1–60.9)	49.4% (38.8–60)	53.8% (43.6–63.9)	46.6% (36.4–57)	1.018 (0.566–1.832)
Total	91	88	179					

IPF, idiopathic pulmonary fibrosis; PPV, positive predictive value; NPV, negative predictive value

### Comparison between IPF and non-IPF

One-year FVC changes showed significant differences between IPF and non-IPF (-138.6 mL versus 18.2 mL, p = 0.014). One-year outcomes were not significantly different between IPF and non-IPF (p = 0.079) (Table 4).

**Table 4:** One-year outcomes.

	IPF (n = 84)	Non-IPF (n = 75)	p value
Improved	6	14	0.079
Unchanged	53	44	
Deteriorated	25	17	
Expired	5	2	

“Deteriorated” was defined as any of the following: > 10% relative decline in FVC, lung transplantation, or death, and “improved” was defined as > 10% relative improvement in FVC.

Seven patients that expired within one year are included in “Deteriorated”.

Twelve patients with IPF and 15 patients with non-IPF did not undergo follow-up pulmonary function testing.

**Fig 1:**
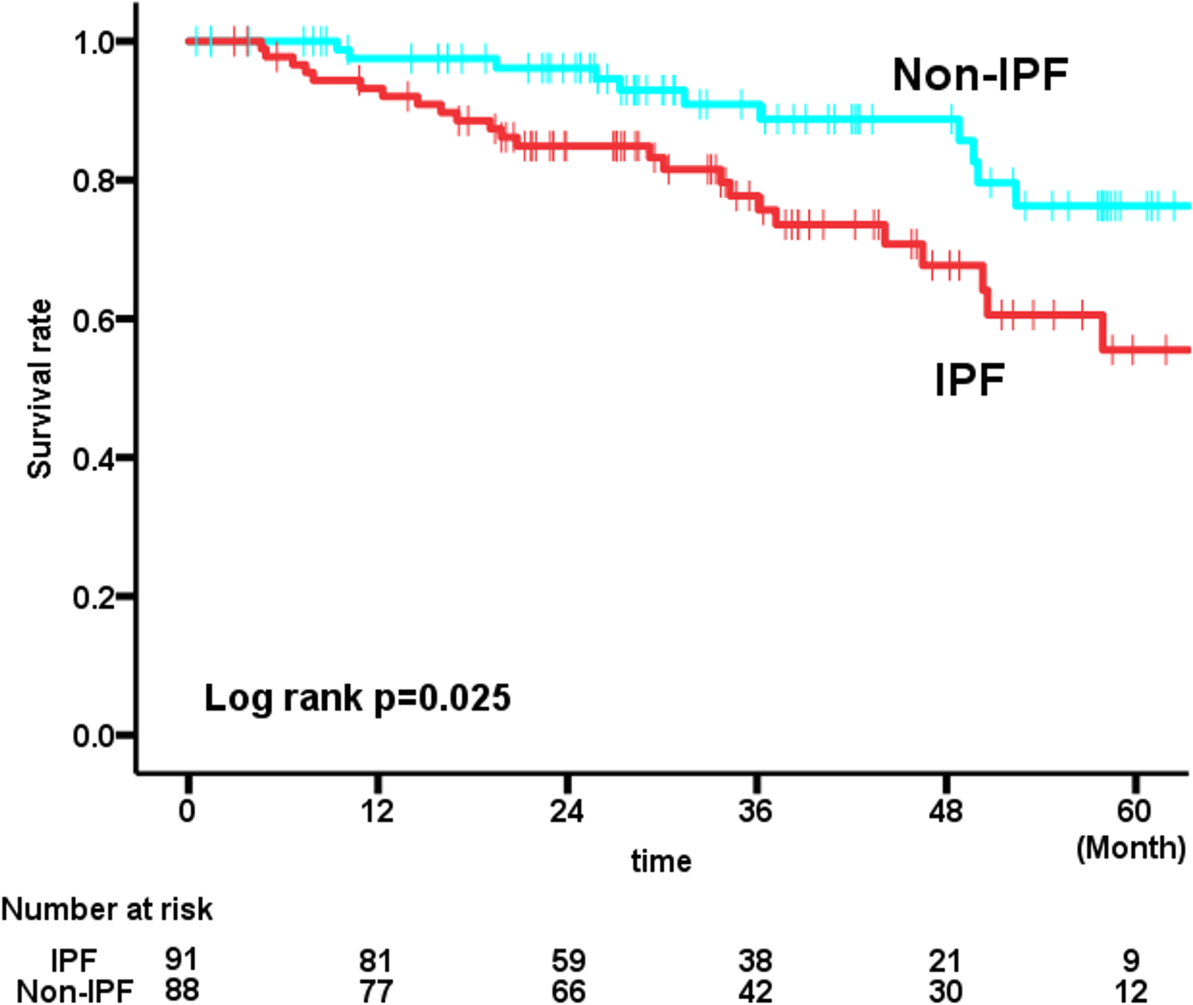
Survival of IPF and non-IPF patients with possible UIP pattern on HRCT. Among patients with possible UIP pattern on HRCT, those with IPF had significantly worse survival than those with non-IPF (Log-rank test, p = 0.025).

Patients with IPF had a significantly worse prognosis than those with non-IPF after adjustment for age and gender [HR = 2.127, 95%CI (1.037–4.362) p = 0.039]. The Kaplan-Meire survival curves showed patients with IPF had a significantly worse prognosis than those with non-IPF (Logrank test, p = 0.025) (Fig 1). Similar results were observed for the initial diagnosis. Patients with IPF had a significantly worse prognosis than those with unclassifiable IIP with a broader definition of pathological UIP pattern after adjustment for age and gender [HR = 3.771, 95%CI (1.104–12.883) p = 0.034].

### Prognostic factors in patients with possible UIP pattern on HRCT

Univariate Cox regression analysis showed that cough, modified MRC score, percent predicted FVC, percent predicted diffusing capacity of lung for carbon monoxide (DLco), and diagnosis of IPF were significant prognostic factors (Table 5). Multivariate Cox regression analysis with adjustment for age and gender demonstrated that low %FVC (HR, 0.969; 95% CI, 0.952–0.987; p = 0.001), high modified MRC score (HR, 1.893; 95% CI, 1.278–2.804; p = 0.001), and diagnosis of IPF (HR, 3.161; 95% CI, 1.443–6.922; p = 0.004) were independent prognostic factors. The results in Table 6 show that Harrell’s C values increased after adding more confounders. The Harrell C values of model 2 (%FVC, IPF, age, and gender) were higher than those of model 1 (%FVC, age, and gender). Moreover, the Harrell C values of model 3 (%FVC, IPF, MMRC, age, and gender) were higher than those of model 2.

**Table 5:** Unadjusted and adjusted Cox Models for survival by selected measures at study baseline.

	Crude			Adjustment for age and gender		
	HR (95% CI)	p	p<0.1	HR (95% CI)	p	p<0.1
Age, y.o.	1.018 (0.977–1.061)	0.391				
Gender, male	0.837 (0.58–1.208)	0.342				
Smoking, never		0.497			0.332	
, ex	1.149 (0.577–2.288)	0.693		0.718 (0.294–1.757)	0.469	
, current	0.48 (0.109–2.116)	0.332		0.296 (0.059–1.481)	0.138	
Cough, yes	0.609 (0.392–0.945)	0.027	*	0.548 (0.349–0.859)	0.009	*
Modified MRC	2.643 (1.799–3.883)	<0.001	*	2.658 (1.823–3.876)	<0.001	*
FVC, % of predicted	0.969 (0.955–0.983)	<0.001	*	0.965 (0.951–0.98)	<0.001	*
DLCO, % of predicted	0.98 (0.963–0.996)	0.014	*	0.974 (0.959–0.99)	0.001	*
FEV_1_‎/FVC, %	1.067 (1.023–1.113)	0.003	*	1.078 (1.032–1.126)	0.001	*
PaO_2_, Torr	0.986 (0.955–1.017)	0.363		0.985 (0.955–1.016)	0.352	
IPF, yes	2.174 (1.085–4.357)	0.029	*	2.127 (1.037–4.362)	0.039	*
BAL: Macrophages, %	1.019 (0.994–1.045)	0.144		1.017 (0.991–1.044)	0.207	
Neutrophils, %	0.971 (0.937–1.006)	0.099	*	0.972 (0.938–1.008)	0.126	
Lymphocytes, %	1.014 (0.965–1.066)	0.579		1.019 (0.974–1.065)	0.420	
Eosinophils, %	0.97 (0.834–1.13)	0.698		0.969 (0.827–1.134)	0.690	

MRC, Medical Research Council scale; FVC, forced vital capacity; DLCO, diffusing capacity for carbon monoxide; FEV1, forced expiratory volume in one second; IPF, idiopathic pulmonary fibrosis; BAL, bronchoalveolar lavage

* yes

**Table 6:** Multivariate Cox hazard analysis for all-cause mortality with adjustment for age and gender.

Variables	HR (95% CI)	p value	C-statistics
FVC, %	0.969 (0.952–0.987)	0.001	0.814
IPF, yes	3.161 (1.443–6.922)	0.004	
Modified MRC	1.893 (1.278–2.804)	0.001	

FVC, forced vital capacity; IPF, idiopathic pulmonary fibrosis; MRC, Medical Research Council scale

## Discussion

In this retrospective study, we studied a total of 179 ILD patients with a surgical lung biopsy and a possible UIP pattern on HRCT. Non-IPF was identified in about a half of the patients with possible UIP pattern on HRCT. Patients with IPF diagnosed by MDD have more disease progression and worse prognosis than those with non-IPF. Multivariate analysis demonstrated that the diagnosis of IPF, high modified MRC score, and low %FVC were independent significant prognostic factors in our cohort. These results indicate that a possible UIP pattern on HRCT did not necessarily indicate a diagnosis of IPF, and patients with IPF diagnosed through MDD with SLB have worse prognosis than those with an alternative diagnosis. We reaffirmed that multidisciplinary discussion is crucial in patients with possible UIP pattern on HRCT.

Our study cohort consisted of 179 SLB patients with possible UIP pattern on HRCT from 10 Japanese hospitals that have experts in the field of ILD. This is the largest cohort to date of consecutive patients with possible UIP pattern on HRCT who underwent SLB. This cohort also had a similar gender and age distribution, and despite some mild pulmonary function impairment compared with other cohorts [10,12,13]. Because populations without definite diagnosis and without known causes of ILD such as CTD or CHP before SLB reflect the real world, we believe our cohort is suitable for diagnostic evaluations.

A variety of diseases other than IPF, such as unclassifiable IIP, idiopathic NSIP, CHP, and CTD-ILD were observed in our cohort. In addition, significant differences were observed in the disease progression and the prognosis between IPF and non-IPF. These results support the importance of a diagnosis of IPF in this population. Because SLB is occasionally difficult to carry out for a variety of reasons, such as poor pulmonary function testing, severe fibrotic changes, and patient unwillingness because of the invasive nature and associated risks, consideration should be given to the differential diagnosis in patients with a possible UIP pattern on HRCT. Because patients with non-IPF showed significantly higher % lymphocytes in BALF than those with IPF, lymphocytosis in BALF may suggest a diagnosis of non-IPF.

A quarter of patients were diagnosed with unclassifiable IIPs in this study, which is compatible with previous studies that reported unclassifiable IIPs in approximately 10 to 25% of all patients with ILD [16]. The reason for the high prevalence of unclassifiable IIP in our cohort may have been due to adherence to strict guideline criteria. The high prevalence of unclassifiable ILD in patients with SLB highlights the need for a broader consensus on how to diagnose fibrotic ILDs. In the recent perspective cited above, a standardized ontological framework for the classification of fibrotic ILD was proposed [16]. Because unclassifiable IIPs have attracted attentions recently [15,16,17,18,19], further studies are needed to evaluate the reasons for and impacts of unclassifiable IIPs.

Our study did not demonstrate a strong association between a possible UIP pattern on HRCT and a diagnosis of IPF, which contradicts a few recent reports [10,12,13]. We offer these possible explanations. In Fell’s study, patients with unclassifiable IIPs were excluded from the cohort [10]. Considering that a quarter of patients with possible UIP pattern on HRCT were diagnosed as unclassifiable IIPs in the present study, exclusion of unclassifiable IIPs in their cohort might have resulted in under-evaluation of the possibility of non-IPF. Analysis of cohorts from other recent randomized controlled studies in IPF showed that a possible UIP pattern on HRCT has a high PPV for the presence of a pathological pattern of UIP [12,13]. Based on their findings, those authors concluded that a possible UIP pattern on HRCT is sufficient to make a diagnosis of IPF in clinically appropriate settings. However, all one can really conclude from this is that the PPV of a possible UIP pattern on HRCT in predicting pathological UIP is high in a patient population already diagnosed with IPF by a local physician based on SLB [20]. Indeed, a recent paper reported that about 60% of patients with possible UIP pattern on HRCT had a diagnosis of IPF in the UCSF cohort, which is similar to the results of our study [14]. In their study, the addition of age, sex and total traction bronchiectasis score improved PPV for a diagnosis of IPF [14]. Further studies are needed to define clinically appropriate settings that allow the differentiation of IPF in patients with a possible UIP pattern on HRCT.

Although age and sex were predictive factors for IPF in another cohort [10], they are not so in the present study. The reasons for this discrepancy are unclear. Salisbury et al. reported that age and gender were not predictors of IPF in their cohort in the absence of radiologic honeycombing [21], which might support our findings. Another possibility is that since all patients in this study underwent SLB, patients who were elderly and more likely to have diagnosis of other than IPF might have been included in the study. In addition, all patients in this study were Japanese, so population and racial difference might have influenced the results. Further studies will be needed to elucidate the impacts of age and sex on the diagnosis of IPF.

There are several limitations in this study. First, the diagnoses examined in this study were made in individual hospitals; therefore, there may be some biases in diagnosis between hospitals. However, we suppose this is less likely because all participating hospitals had experts in the field of ILD, which helps to ensure the accuracy of diagnostic evaluations. In addition, the finding in a recent study that agreement in MDD diagnosis of expert groups is acceptable in IPF [22] may lend support to the validity of our study. Second, all the patients underwent SLB, so considerably selected cases are assumed to have been included. However, we suppose this is an inevitable limitation in all studies of patients with SLB. Third, since this was a retrospective study, we could not evaluate an important variable of HRCT possible UIP pattern with a total traction bronchiectasis score that contributed to diagnostic accuracy. Finally, the diagnosis in MDD was not standardized in the study. Therefore, diagnostic differences among the attended hospitals may exist and affect the results especially in the diagnosis of unclassifiable IIP. Further standardized studies or central evaluations are needed to study the diagnostic agreement.

In conclusion, we studied the frequency of IPF and prognostic factors in a biopsy-proven ILD cohort of patients with a possible UIP pattern on HRCT. Although previous studies have reported high positive predictive values of possible UIP patterns on HRCT for predicting IPF, this study demonstrated that patients with possible UIP patterns on HRCT did not necessarily have IPF; the PPV for IPF in patients with possible UIP pattern on HRCT over 65 y.o. was only 51%. Because patients with IPF had a worse prognosis than those with an alternative diagnosis, identification of IPF in patients with a possible UIP pattern on HRCT is crucial. Further studies are needed to define clinically appropriate predictors for IPF.

## Supporting information

S1 File Table A in S1 File. Positive predictive value, negative predictive value, sensitivity, and specificity when classifying patients with IPF based on being at least as old as the age indicated.
Table B in S1 File. Positive predictive value, negative predictive value, sensitivity, and specificity when classifying patients with broader definition of histological UIP pattern based on being at least as old as the age indicate.
Table C in S1 File. The reasons for, broader definition of pathologic UIP pattern in, and most suspected diagnosis of unclassifiable IIP.
Fig A in S1 File. Relationships between age and diagnosis.
(DOCX)

S1 Datasheet Baseline data of the study population (n = 179). (XLSX)

## Abbreviations

BALF: bronchoalveolar lavage fluid
CHP: chronic hypersensitivity pneumonitis
CI: confidence interval
CTD: connective tissue disease
DLco: Diffusion capacity for carbon monoxide
FVC: forced vital capacity
HR: hazard ratio
HRCT: high-resolution CT
IIPs: idiopathic interstitial pneumonias
ILD: interstitial lung disease
IPF: idiopathic pulmonary fibrosis
MDD: multidisciplinary discussion
MRC: Medical Research Council
NSIP: non-specific interstitial pneumonia
PPVs: positive predictive values
SLB: surgical lung biopsy
UIP: usual interstitial pneumonia
