# Supplementary material for: Clinical spectrum and prognostic factors of possible UIP pattern on high-resolution CT in patients who underwent surgical lung biopsy
Source: PLoS One. 2018 Mar 28;13(3):e0193608. doi: 10.1371/journal.pone.0193608 (PMC5873997; doi:10.1371/journal.pone.0193608)
Supplement: S1 File — Table A in S1 File. Positive predictive value, negative predictive value, sensitivity, and specificity when classifying patients with IPF based on being at least as old as the age indicated. Table B in S1 File. Positive predictive value, negative predictive value, sensitivity, and specificity when classifying patients with broader definition of histological UIP pattern based on being at least as old as the age indicate. Table C in S1 File. The reasons for, broader definition of pathologic UIP pattern in, and most suspected diagnosis of unclassifiable IIP. Fig A in S1 File. Relationships between age and diagnosis. (DOCX) [file pone.0193608.s001.docx]

**Supporting Information: S1 File**

**Table A. The reasons for, broader definition of pathologic UIP pattern in, and most suspected diagnosis of unclassifiable IIP**

| Case | Age | Sex | Inadequate clinical, radiologic, or pathologic data | Previous therapy resulting in substantial alteration | New entity, or unusual variant of recognized entity | Multiple HRCT and/or pathologic patterns | Broader definition of pathologic UIP pattern | Most suspected diagnosis |
| --- | --- | --- | --- | --- | --- | --- | --- | --- |
| 1 | 59 | male | No | No | No | Yes | Yes | IPF |
| 2 | 67 | male | No | No | No | Yes | Yes | CTD-ILD |
| 3 | 75 | male | No | No | Yes | No | No | COP |
| 4 | 78 | male | No | No | Yes | No | No | CHP |
| 5 | 72 | male | No | No | Yes | Yes | No | indeterminate |
| 6 | 70 | female | No | No | No | Yes | Yes | CTD-ILD |
| 7 | 64 | male | No | No | No | Yes | Yes | NSIP |
| 8 | 62 | male | No | No | No | Yes | Yes | CHP |
| 9 | 70 | female | No | No | No | Yes | Yes | CHP |
| 10 | 74 | female | No | No | No | Yes | Yes | CTD-ILD |
| 11 | 71 | male | No | No | Yes | Yes | Yes | CHP |
| 12 | 67 | male | No | No | No | No | Yes | CTD-ILD |
| 13 | 68 | male | No | No | No | Yes | No | NSIP |
| 14 | 65 | male | No | No | No | Yes | Yes | CHP |
| 15 | 73 | female | No | No | No | Yes | Yes | CTD-ILD |
| 16 | 52 | male | No | No | No | Yes | Yes | CHP |
| 17 | 64 | male | No | No | No | Yes | Yes | SR-ILD |
| 18 | 68 | female | No | No | No | Yes | Yes | CTD-ILD |
| 19 | 67 | male | No | No | No | Yes | Yes | CHP |
| 20 | 63 | male | No | No | No | Yes | No | NSIP |
| 21 | 74 | male | No | No | No | Yes | Yes | CHP |
| 22 | 48 | male | No | No | No | Yes | Yes | CHP |
| 23 | 67 | female | No | No | No | Yes | Yes | CHP |
| 24 | 73 | male | No | No | No | Yes | No | NSIP |
| 25 | 52 | male | No | No | No | Yes | Yes | IPF |
| 26 | 61 | male | No | No | No | Yes | Yes | IPF |
| 27 | 70 | male | No | No | No | Yes | Yes | IPF |
| 28 | 40 | female | No | No | No | Yes | Yes | CHP |
| 29 | 53 | male | No | No | No | Yes | Yes | IPF |
| 30 | 76 | female | No | No | No | Yes | Yes | CHP |
| 31 | 61 | female | No | No | No | Yes | Yes | IPF |
| 32 | 61 | male | No | No | No | Yes | Yes | IPF |
| 33 | 55 | male | No | No | No | Yes | Yes | IPF |
| 34 | 72 | male | No | No | No | Yes | Yes | CTD-ILD |
| 35 | 71 | male | No | No | No | Yes | Yes | IPF |
| 36 | 65 | male | Yes | No | No | Yes | Yes | SR-ILD |
| 37 | 58 | male | Yes | No | No | No | Yes | CHP |
| 38 | 59 | female | Yes | No | No | No | Yes | CHP |
| 39 | 73 | female | No | No | No | Yes | Yes | IPF |
| 40 | 44 | male | No | No | No | Yes | Yes | CHP |
| 41 | 69 | female | No | No | No | Yes | Yes | CHP |
| 42 | 65 | male | No | No | No | Yes | Yes | IPF |
| 43 | 54 | male | No | Ｎｏ | Ｎｏ | Ｙｅｓ | Yes | IPF |
| 44 | 72 | male | No | No | No | No | Yes | CHP |
| 45 | 68 | female | No | No | No | No | Yes | CHP |
| 46 | 75 | male | No | No | Yes | No | No | NSIP |
| 47 | 50 | female | No | No | No | No | Yes | CTD-ILD |

UIP, usual interstitial pneumonia; IIP, idiopathic interstitial pneumonia; HRCT, high resolution CT; IPF, idiopathic pulmonary; CTD-ILD, connective tissue disease-interstitial lung disease; COP, cryptogenic organizing pneumonia; CHP, chronic hypersensitivity pneumonia; NSIP, nonspecific interstitial pneumonia

**Table B. Positive predictive value, negative predictive value, sensitivity, and specificity when classifying patients with IPF based on being at least as old as the age indicated.**

| Age | IPF | Non-IPF | Total | PPV | NPV | Sensitivity | Specificity | Odds |
| --- | --- | --- | --- | --- | --- | --- | --- | --- |
| -44 | 3 | 3 | 6 | 50% (15.6-84.4) | 49.1% (41.7-56.6) | 3.3% (0.8-8.3) | 96.6% (91.4-99.1) | 0.966 (0.19-4.919) |
| 45-49 | 1 | 2 | 3 | 33.3% (2.3-83.9) | 48.9% (41.5-56.2) | 1.1% (0.1-4.7) | 97.7% (93.1-99.6) | 0.478 (0.043-5.365) |
| 50-54 | 5 | 9 | 14 | 35.7% (14.6-61.7) | 47.9% (40.3-55.5) | 5.5% (2-11.4) | 89.8% (82.3-94.9) | 0.51 (0.164-1.588) |
| 55-59 | 8 | 11 | 19 | 42.1% (22-64.2) | 48.1% (40.5-55.9) | 8.8% (4.1-15.7) | 87.5% (79.5-93.3) | 0.675 (0.258-1.766) |
| 60-64 | 25 | 16 | 41 | 61% (45.7-74.9) | 52.2% (43.9-60.4) | 27.5% (19-37.2) | 81.8% (72.9-88.9) | 1.705 (0.837-3.47) |
| 65-69 | 26 | 23 | 49 | 53.1% (39.2-66.6) | 50% (41.5-58.5) | 28.6% (20-38.4) | 73.9% (64.1-82.3) | 1.13 (0.585-2.183) |
| 70-74 | 18 | 17 | 35 | 72% (52.8-86.9) | 52.6% (44.7-60.4) | 19.8% (12.5-28.7) | 92% (85.2-96.5) | 2.853 (1.127-7.221) |
| 75- | 5 | 7 | 12 | 41.7% (17.5-69.1) | 48.5% (41-56.1) | 5.5% (2-11.4) | 92% (85.2-96.5) | 0.673 (0.205-2.205) |
| Total | 91 | 88 | 179 |  |  |  |  |  |

IPF, idiopathic pulmonary fibrosis; PPV, positive predictive value; NPV, negative predictive value

**Table C. Positive predictive value, negative predictive value, sensitivity, and specificity when classifying patients with broader definition of histological UIP pattern based on being at least as old as the age indicated.**

|  | Broader definition of pathological UIP pattern* | |  |  |  |  |  |  |
| --- | --- | --- | --- | --- | --- | --- | --- | --- |
| Age | Yes | No | Total | PPV | NPV | Sensitivity | Specificity | Odds |
| -54 | 17 | 6 | 23 | 73.9% (54.1-88.7) | 26.9% (20.4-34.2) | 13.0% (8.0-19.4) | 87.5% (76.3-94.8) | 1.044 (0.386-2.825) |
| 55-65 | 54 | 20 | 74 | 73.0% (62.2-82.2) | 26.7% (18.8-35.6) | 41.2% (33.0-49.8) | 58.3% (44.2-71.6) | 0.982 (0.502-1.921) |
| 65- | 60 | 22 | 82 | 73.2% (63.0-81.9) | 26.8% (18.7-36.1) | 45.8% (37.4-54.4) | 54.2% (40.1-67.8) | 0.999 (0.514-1.939) |
| Total | 131 | 48 | 179 |  |  |  |  |  |

*: A broader pathologic UIP pattern was defined as a UIP pattern and additional pathologic patterns suggestive but not definitive for a diagnosis of CTD (e.g., lymphoid aggregates with germinal center and/or prominent plasmacytic infiltration) or CHP (e.g., centrilobular and/or bridging fibrosis), or a pattern in which differentiation between a UIP pattern and a NSIP pattern was difficult.

UIP, usual interstitial pneumonia; PPV, positive predictive value; NPV, negative predictive value

**Fig A. Relationships between age and diagnosis**


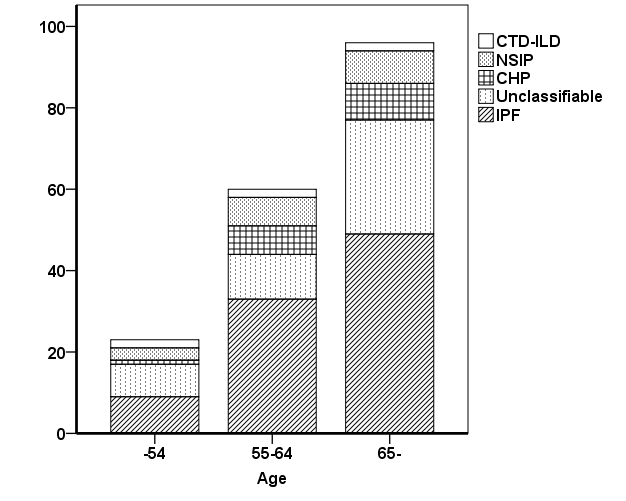


IPF, idiopathic pulmonary fibrosis; IIPs, idiopathic interstitial pneumonias; NSIP, non-specific interstitial pneumonia; CHP, chronic hypersensitivity pneumonitis; CTD, connective tissue disease; ILD, interstitial lung disease
